# Relating Lateralization of Eye Use to Body Motion in the Avoidance Behavior of the Chameleon (*Chamaeleo chameleon*)

**DOI:** 10.1371/journal.pone.0070761

**Published:** 2013-08-14

**Authors:** Avichai Lustig, Hadas Ketter-Katz, Gadi Katzir

**Affiliations:** 1 Department of Neurobiology and Ethology, University of Haifa, Haifa, Israel; 2 Department of Environmental and Evolutionary Biology, University of Haifa, Haifa, Israel; 3 Department of Marine Biology, University of Haifa, Haifa, Israel; 4 Department of Biology, University of Haifa at Oranim, Tivon, Israel; University of Sussex, United Kingdom

## Abstract

Lateralization is mostly analyzed for single traits, but seldom for two or more traits while performing a given task (e.g. object manipulation). We examined lateralization in eye use and in body motion that co-occur during avoidance behaviour of the common chameleon, *Chamaeleo chameleon*. A chameleon facing a moving threat smoothly repositions its body on the side of its perch distal to the threat, to minimize its visual exposure. We previously demonstrated that during the response (i) eye use and body motion were, each, lateralized at the tested group level (N = 26), (ii) in body motion, we observed two similar-sized sub-groups, one exhibiting a greater reduction in body exposure to threat approaching from the left and one – to threat approaching from the right (left- and right-biased subgroups), (iii) the left-biased sub-group exhibited weak lateralization of body exposure under binocular threat viewing and none under monocular viewing while the right-biased sub-group exhibited strong lateralization under both monocular and binocular threat viewing. In avoidance, how is eye use related to body motion at the entire group and at the sub-group levels? We demonstrate that (i) in the left-biased sub-group, eye use is not lateralized, (ii) in the right-biased sub-group, eye use is lateralized under binocular, but not monocular viewing of the threat, (iii) the dominance of the right-biased sub-group determines the lateralization of the entire group tested. We conclude that in chameleons, patterns of lateralization of visual function and body motion are inter-related at a subtle level. Presently, the patterns cannot be compared with humans' or related to the unique visual system of chameleons, with highly independent eye movements, complete optic nerve decussation and relatively few inter-hemispheric commissures. We present a model to explain the possible inter-hemispheric differences in dominance in chameleons' visual control of body motion during avoidance.

## Introduction

Lateralization [1] of cognitive and of motor functions in vertebrates has been extensively documented, with numerous examples pertaining to vision and visually guided behavior [2]. At the behavioral level, studies of lateralization have focused on limb use [3]–[5], a sensory modality or organ (e.g., eye) [6]–[10] or the entire body (i.e. body orientation) [11], [12]. However, testing for lateralization of more than one aspect while performing a given behavior is uncommon [13]. For example, in animals tested on the task of a visually guided manipulation of an object, analyses have focused on either eye use or limb use but not on both. This leaves open the question: are patterns of lateralization of limb use and of eye use related?

In addressing visually guided behavior, one should consider the animal's morphology. Fish and amphibians have limited movement of the head relative to the torso so that the direction of monocular viewing of a target closely matches the direction of the ipsilateral body side [14]–[17]. In terms of limb use, there are reports of lateralization of fin use in fish [13], while amphibians show lateralization of the forelimb in locomotion, facial wiping and swallowing of prey [3], [18], [19]. Most reptiles have relatively restricted eye movements yet well-developed necks so that gaze direction is by head movements. While lateralization of eye use has been reported in reptiles [12], [20], [21] there are no reports on limb use. In common with ectotherms, birds how laterally placed eyes with relatively restricted movements [22], [23] and weak inter-hemispheric connections [24]. Birds' restricted eye movements are compensated for by exceptionally long and flexible necks that allow a wide range of head orientations. Lateralization of eye use in birds has been well documented and, in many species, there is an extensive use of the feet in visually guided behavior patterns such as ground scratching, food grasping and climbing [25]–[28]. However, examples of lateralized eye use that co-occur with limb use, in the performance of a given task are, to our knowledge, lacking. An example would be to test for lateralization in eye use and in feet use in a crow that is making a tool: Is there a preferred foot for manipulation and a preferred eye for viewing the manipulation?

Common chameleons (*Chamaeleo chameleon*) are arboreal lizards that exhibit highly independent, large-amplitude, eye movements [29] which allow them to rapidly alternate between monocular and binocular viewing of targets [30]–[32]. While their movements are typically slow, chameleons perform rapid and highly precise body-position corrections while avoiding a threat [33], [34]. Upon the approach of a threat (e.g., a predator or a human), a chameleon will change its position on a perch so as to keep its body on the side of the perch distal to the threat, thus, minimizing its body exposure. If the threat moves in a given direction, the chameleon will synchronously counter-rotate smoothly and rapidly.

We previously demonstrated that during avoidance, eye use [33] is lateralized under binocular but not under monocular viewing of the threat. Also, the patterns of body motion and thus of body exposure, were lateralized at the tested group level (N = 26). A further analysis revealed two similar-sized sub-groups in terms of body motion [34]: One exhibited significantly lower exposure (and thus better concealment) to a threat approaching from the right (termed “right-biased sub-group”) and the other – to a threat approaching from the left (termed “left-biased sub-group”). This lateralization of body exposure was observed under conditions that allowed only monocular or both monocular and binocular viewing of the threat [33], [34].

In ectotherms and birds, the optic nerves are highly decussated, having few or no ipsilateral projections [2], [20], [32]. Also, in these groups, inter-hemispheric commissures are relatively few and small compared with mammals inter-hemispheric information transfer may well be less efficient [24]. Because of the greater separation between hemispheres it is therefore expected that ectotherms will show a greater correspondence between lateralization of hemispheric functions (as expressed in body motion) and lateralization of eye use.

Using chameleons as a model, we ask: How are the patterns of eye use and of body motion related to each other in the performance of a distinct visually guided behavior (the avoidance response)? How are the relationships expressed at the sub-group level? Are patterns of lateralization of eye use and of body motion similar to those in mammals?

## Materials and Methods

The research was conducted at the Department of Biology, University of Haifa, Oranim Campus in Tivon, Israel, between November 2006 and November 2009. Collection, maintenance and experimentation with the experimental animals (common chameleons, *Chamaeleo chameleon*) were performed under permits from the Israeli Nature and Parks Authority (permit 2011/11411) and were specifically approved by the University of Haifa ethics committee (permit 095/08). Methods are provided here in brief; further details can be found elsewhere [33].

### Experimental Setup

**Figure 1:**
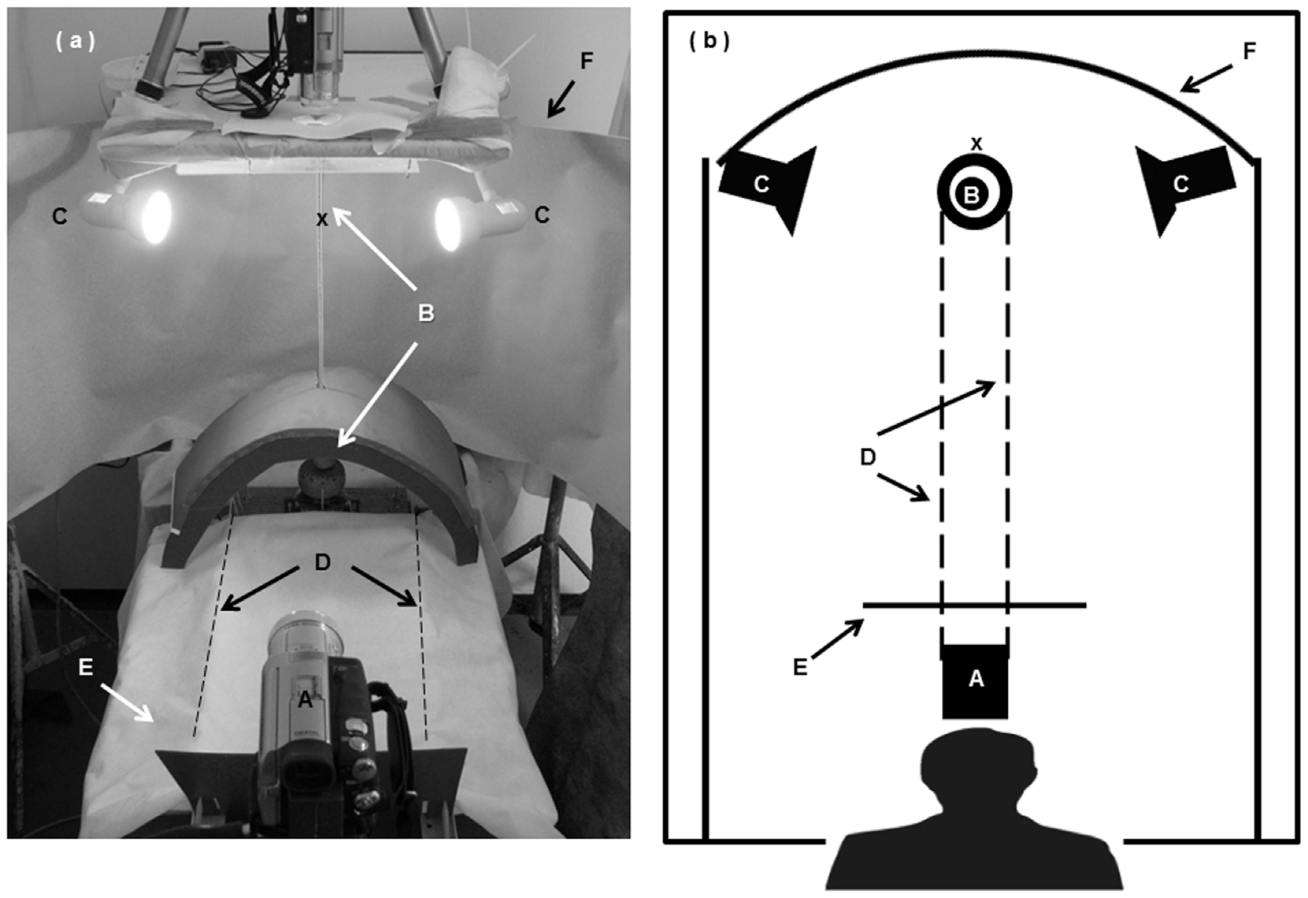
The experimental setup (a – oblique view; b – schematic overhead view). The experimenter, positioned behind the camera (A), acts as the threat stimulus. Chameleon (X); vertical, rotatable pole (B); incandescent lights (C); pole rotation cords (D); visual barrier (E); screen (F). Reprinted from [33] under a CC BY license, with permission from Springer, original copyright 2012.

The experimental apparatus (Fig. 1) was built to allow the controlled motion of a chameleon on a perch. It comprised a vertical, 80-cm long pole that could be rotated (clockwise or anti-clockwise) manually using 2 thin cords. Two 60 W incandescent bulbs in hoods illuminated the pole from both sides. The experimenter, positioned 1.2 m from the pole, was also the threatening stimulus. A video camera was positioned in front of the experimenter, pointing horizontally at the pole at the level of the subject's position. Pole diameter (“narrow” or “wide”) was determined in relation to the chameleon's head width. Narrow pole diameters ranged between 2 mm and 4 mm, while wide pole diameters ranged between 4 mm and 14 mm. The mean widths of the tested chameleons' heads ranged between 4.32±0.01 mm and 11.18±0.64 mm (mean ± SD). A narrow pole allowed the tested chameleon to view the threat binocularly, while a wide pole allowed only monocular viewing. The experimental apparatus was surrounded on three sides by a 2 m-high white cloth screen. To enhance the image contrast required for computer analysis of the videos, the pole was painted pink and the background gray.

### Testing Procedures

A chameleon to be tested was taken by hand from its cage and allowed to grasp the pole; the experimenter then positioned himself at the distal side of the experimental table. Once the chameleon had settled, the pole was rotated 30° (at ∼15°/s) in a given direction (Phase 1, approx. 2 s) and then held stationary, allowing the chameleon to respond (Phase 2, approx. 8 s, see Fig. 6 in [34]). This was repeated 3 times in succession in the same direction. Rotating the pole manually ensured smooth turning without compromising the chameleon's foothold due to centrifugal force.

Following each pole rotation, the chameleon corrected its position relative to the threat and the subsequent rotation was carried out only after a “steady state” had been reached, i.e., with minimal or no apparent body rotation observed. The full sequence of a single pole rotation plus the chameleon's response was termed a run. Three consecutive runs composed a test. Mean test duration was 9.75 s.

Each chameleon (N = 26) was tested once with a left-approaching threat and once with a right-approaching threat, for each given pole width. When possible, the chameleons were tested once again at a different age. This applied to 15 of the tested individuals.

The choice of direction of the first rotation, for poles of each width, was based on a table of random numbers. The following rotation was in the opposite direction. Inter-test interval was 5 min.

The video sequences were edited using Adobe Elements™ software and processed using a specially written program (SIPL Lab, Technion, Israel) for eye motion analysis. Tests over all ages (from 1 day post-hatch to 1 year of age) were pooled for the statistical analysis to obtain a clear picture of how lateralization is expressed in the entire group. In the pooled data, each individual was represented once for every viewing condition (monocular or binocular) and for every direction of threat approach. If an individual was tested at two different ages, an average was calculated for the two parameters analyzed (durations in viewing categories & shifts between viewing categories, see below), for the data of both ages.

**Figure 2:**
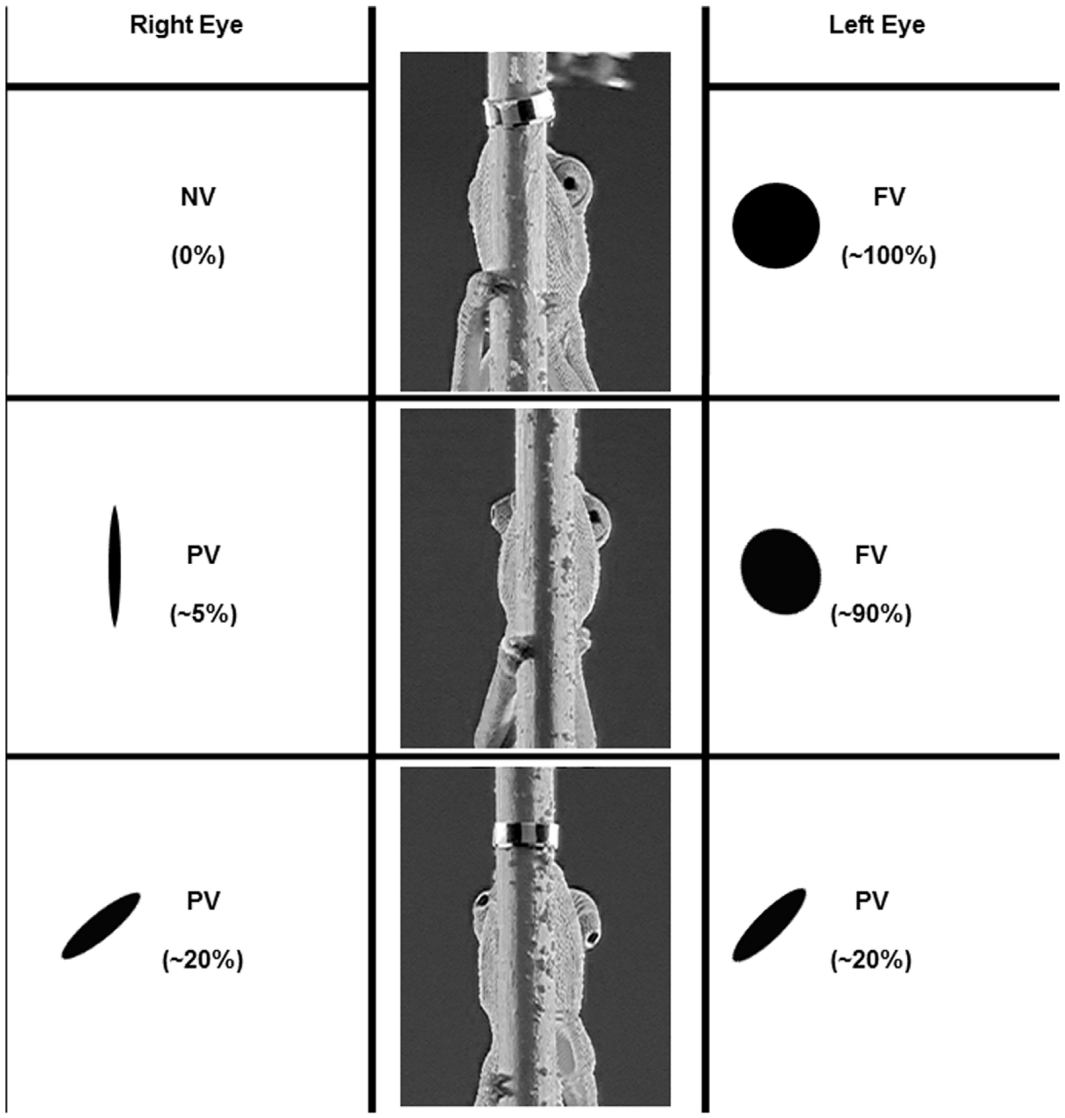
The range of eye apertures for the different viewing categories. Frontal viewing (FV), from circular to oval-shaped eye opening (respectively ca. 100% to ca. 20% of the maximum). Peripheral viewing (PV), from oval to slit-shaped eye opening (respectively ca. 20% to ca. 0%). Not visible (NV), eye opening cannot be observed. Reprinted from [33] under a CC BY license, with permission from Springer, original copyright 2012.

Eye motion was analyzed using a semi-automated recording of eye direction in the video sequences. The direction of viewing of a given eye, as captured by the video camera, was determined by the observer. The “visual sphere” around each eye was divided into three subsections, based on the observed direction of the eye's axis. The direction was estimated from the shape of the eyelid (the aperture formed by the fused eyelids, Fig. 2, and see [35]). An aperture of between a full circle (ca. 100% open) and an oval-shaped opening (ca. 20% of maximal opening) was considered as viewing the threat directly, i.e. parallel to the camera's axis, and was termed frontal viewing (FV). An aperture appearing elliptical or crescent-shaped (ca. 20% to near 0% of maximal opening) was considered as having its viewing axis deviated laterally and/or vertically relative to the camera's axis and was termed peripheral viewing (PV). Under these conditions, the axis of the eye could be estimated but actual viewing of the target was considered impossible (pupil not visible). If an aperture could not be observed (0%) it was termed “not visible” (NV).

### Duration in Viewing Categories

For each sequence analyzed, single video frames were extracted at a 4-frame interval. From each extracted frame, eye viewing category (FV, PV or NV) was recorded manually. The proportion of the duration spent by each eye in each category was calculated from the number of frames in that sequence.

For each run and for each eye, we calculated the ratio of the number of frames recorded for each directional category to the total number of frames sampled for that run. The mean ratio for each viewing direction category was then calculated for the three consecutive runs of each test, providing a mean duration for each directional category of each eye for all tested individuals. The above procedure was performed separately for each pole width and each direction of pole rotation.

Due to the numerous variables analyzed, the statistical p-values of their analyzed results (Table 1) were adjusted using the Bonferroni correction.

**Table 1:** Eye durations (proportions) in the different viewing categories (FV, PV or NV).

Line	Sub-group	Viewing category	Right Approaching Threat	Left Approaching Threat	ANOVA results	Wide Pole	Narrow Pole	ANOVA results
1	**RBG**	**FV**	0.719±0.046	0.83±0.038	F_(1,9)_ = 2.972, p = 0.119	0.778±0.047	0.77±0.023	F_(1,9)_ = 0.026, p = 0.875
2	**RBG**	**PV**	0.149±0.019	0.118±0.015	F_(1,9)_ = 6.517, p = 0.093	0.091±0.014	0.177±0.024	F_(1,9)_ = 15.932, p = 0.009
3	**RBG**	**NV**	0.132±0.053	0.052±0.029	F_(1,9)_ = 1.392, p = 0.268	0.131±0.046	0.053±0.012	F_(1,9)_ = 3.23, p = 0.106
4	**LBG**	**FV**	0.847±0.026	0.866±0.018	F_(1,6)_ = 1.288, p = 0.3	0.856±0.02	0.857±0.034	F_(1,6)_<0.001, p = 0.990
5	**LBG**	**PV**	0.116±0.026	0.079±0.016	F_(1,6)_ = 5.152, p = 0.064	0.069±0.024	0.126±0.031	F_(1,6)_ = 2.195, p = 0.189
6	**LBG**	**NV**	0.037±0.006	0.055±0.015	F_(1,6)_ = 1.429, p = 0.277	0.075±0.014	0.018±0.008	F_(1,6)_ = 14.598, p = 0.027

			Right Approaching Threat	Left Approaching Threat	ANOVA results	Leading Eye	Following Eye	ANOVA results
7	**RBG**	**FV**	0.622±0.038	0.597±0.031	F_(1,13)_ = 0.64, p = 0.438	0.802±0.018	0.417±0.06	F_(1,13)_ = 36.83, p<0.003
8	**RBG**	**PV**	0.169±0.023	0.118±0.017	F_(1,13)_ = 5.394, p = 0.111	0.15±0.018	0.138±0.019	F_(1,13)_ = 0.594, p = 0.455
9	**RBG**	**NV**	0.209±0.04	0.285±0.034	F_(1,13)_ = 3.773, p = 0.074	0.049±0.009	0.445±0.066	F_(1,13)_ = 32, p<0.003
10	**LBG**	**FV**	0.546±0.057	0.551±0.047	F_(1,9)_ = 0.2, p = 0.891	0.768±0.037	0.328±0.07	F_(1,9)_ = 67.155, p<0.003
11	**LBG**	**PV**	0.147±0.022	0.151±0.022	F_(1,9)_ = 0.042, p = 0.841	0.163±0.027	0.135±0.026	F_(1,9)_ = 0.76, p = 0.406
12	**LBG**	**NV**	0.307±0.047	0.298±0.045	F_(1,9)_ = 0.056, p = 0.817	0.068±0.024	0.537±0.076	F_(1,9)_ = 38.901, p<0.003

The results pertain to i) Lines 1–6: the durations as a function of threat approach direction (columns 4–6) and pole width (columns 7–9), ii) Lines 7–12: the durations as a function of threat approach direction (columns 4–6) and eye role (columns 7–9). Provided are ANOVA test results.

### Shifts between Viewing Categories

For each “run”, the frequencies of shifts between viewing categories were analyzed. A “shift” was defined as a change between any two viewing categories (i.e. FV, PV and NV) in consecutive frames. The number of shifts per test provided the frequency. The mean frequencies were used to determine differences between the eyes under different pole widths and directions of rotation.

### Eye Roles

**Figure 3:**
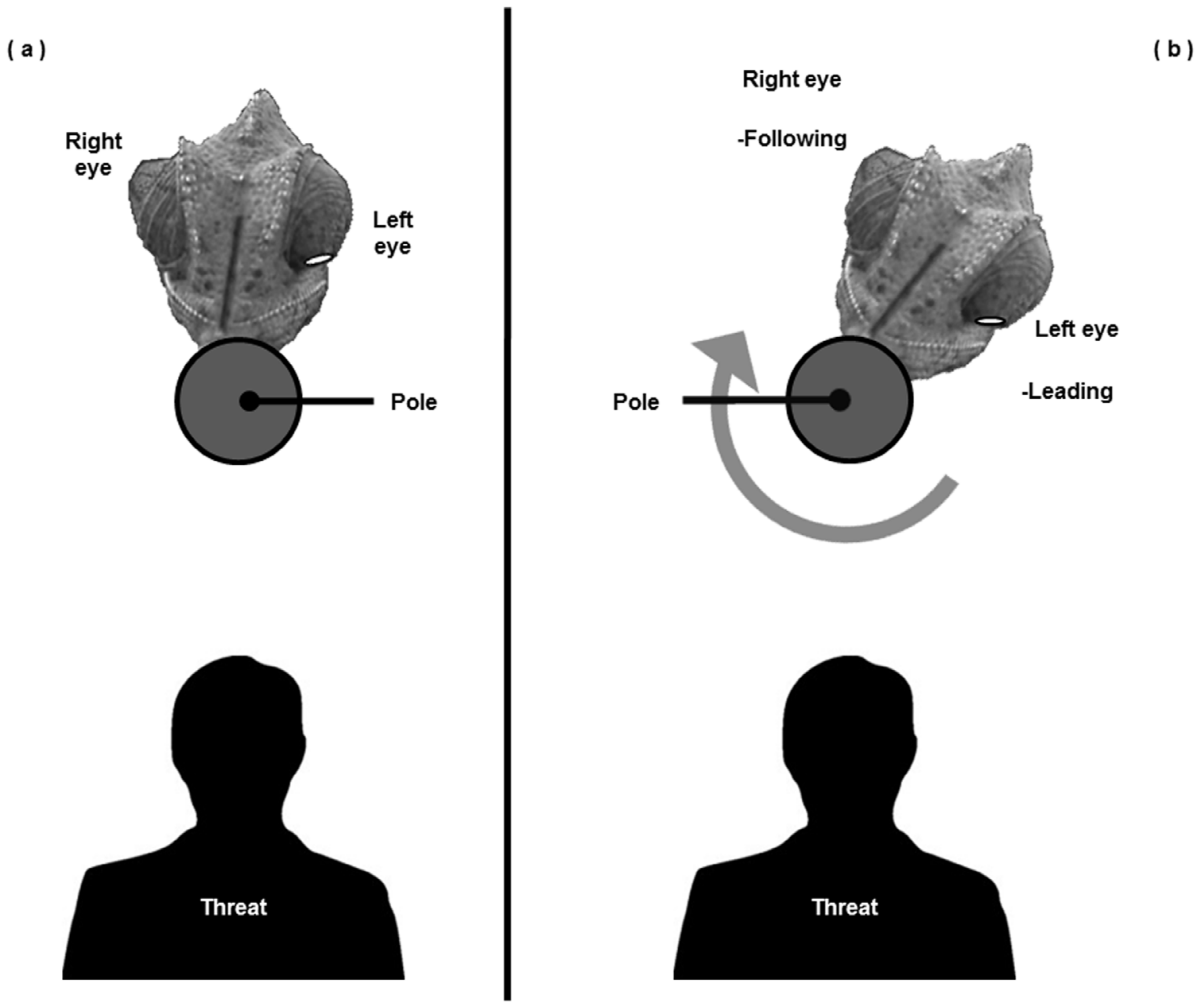
A chameleon on the vertical pole and the threat stimulus (overhead view). (a) The chameleon is positioned opposite (ca.180°) the threat, as an initial state or following a position correction, with its eyes equidistant from the threat. (b) During, or immediately following, pole rotation one eye (the “Leading Eye”) draws closer to the threat while the opposite eye (the “Following Eye”) draws away. When the direction of the pole rotation is reversed, the eye roles are reversed. Reprinted from [33] under a CC BY license, with permission from Springer, original copyright 2012.

Pole rotation resulted in one side of the chameleon approaching the threat and the other moving away from it. The eye on the side approaching the threat in a given test was termed the “leading eye”, while the eye on the side moving away was termed the “following eye” (Fig. 3).

### Statistical Analysis

Analyses of eye use were conducted on data obtained from the same individuals that comprised the sub-groups as reported previously [33], [34]. The analyses were conducted for each eye role (leading or following) separately. Repeated measures MANOVA was used for the right-biased sub-group and for the left-biased sub-group separately, for within-subject analysis. An inter-pole analysis was conducted with data obtained from tests with “wide poles” and with “narrow poles”, while an intra-pole analysis was conducted for tests with “narrow poles” only. This was because (i) the tests on the wide pole comprised data on the leading eyes and not the following eyes. A comparison that included data on the following eyes of the narrow pole tests could only be achieved by performing an intra-pole analysis and (ii) for both sub-groups, the number of subjects tested on the wide pole (N = 10 for the right-biased sub-group and N = 7 for the left-biased sub-group) was lower than the number tested on the narrow pole (N = 14 for the right-biased sub-group and N = 10 for the left-biased sub-group). As the comparisons were within subject, the separate analyses of wide poles and narrow poles allowed a maximal number of narrow-pole subjects.

## Results

### Movements of the Leading Eye as a Function of Threat Approach Direction and of Pole Width

#### Duration in FV

No significant difference was found regarding the FV for both the right-biased or the left-biased sub-groups (Table 1, lines 1,4).

#### Duration in PV

In the right-biased sub-group (N = 10), on a narrow pole, the leading eye spent significantly longer durations in PV compared with that on a wide pole (F_(1,9)_ = 15.932, p = 0.009). No significant difference was found regarding the FV for the left-biased sub-group (Table 1, lines 2,5).

#### Duration in NV

No significant difference was found regarding NV for the right-biased sub-group. In the left-biased sub-group (N = 7), on the narrow pole, the leading eye spent significantly shorter durations in NV than on the wide pole (F_(1,6)_ = 14.598, p = 0.027; Table 1, lines 3,6).

#### Frequency of eye shifts

In the right-biased sub-group (N = 10) the frequencies of eye shifts of the leading eye were significantly higher on the narrow pole than on the wide pole (F_(1,9)_ = 7.139, p = 0.026; Table 2, line 1). No significant differences were found in terms of the frequency of eye shifts in the-left biased sub-group (Table 2, line 2).

**Table 2:** Eye shift frequencies (mean/test) between the three viewing categories (FV, PV or NV).

Line	Sub-group	Right Approaching Threat	Left Approaching Threat	ANOVA results	Wide Pole	Narrow Pole	ANOVA results
1	RGB	2.133±0.371	1.671±0.254	F_(1,9)_ = 1.004, p = 0.343	1.554±0.257	2.25±0.252	F_(1,9)_ = 7.139, p = 0.052
2	LGB	1.625±0.259	1.393±0.216	F_(1,6)_ = 0.616, p = 0.463	1.351±0.233	1.667±0.296	F_(1,6)_ = 0.691, p = 0.438

		Right Approaching Threats	Left Approaching Threats	ANOVA test results	Leading Eye	Following Eye	ANOVA test results
3	RGB	2.619±0.279	1.982±0.22	F_(1,13)_ = 6.706, p = 0.044	1.929±0.234	2.673±0.293	F_(1,13)_ = 6.278, p = 0.052
4	LGB	1.95±0.206	2.192±0.198	F_(1,9)_ = 1.493, p = 0.253	1.742±0.274	2.4±0.302	F_(1,9)_ = 2.075, p = 0.184

The results pertain to i) Lines 1–2: the frequencies as a function of threat approach direction (columns 3–5) and pole width (columns 6–8), ii) Lines 3–4: the frequencies as a function of threat approach direction (columns 3–5) and eye role (columns 6–8). Provided are ANOVA test results.

### Eye Movements as a Function of Threat Approach Direction and Eye Role (narrow pole tests only)

#### Duration in FV

In the right-biased sub-group on a narrow pole (N = 14), the leading eye spent significantly longer durations in FV than the following eye (F_(1,13 )_  = 36.83, p<0.003; Table 1, line 7). The interaction of threat approach direction and eye role was significant (F_(1,13)_ = 15.485, p = 0.006). Consequently, a separate MANOVA was conducted for the leading eye and the following eye, showing that the leading eye spent significantly longer durations in FV under left-approaching threats compared with right-approaching threats (F_(1,13)_ = 22.585, p<0.002; Table 3, line 1). The following eye spent significantly shorter durations in FV under left-approaching vs. right-approaching threats (F_(1,13)_ = 7.944, p = 0.03; Table 3, line 3).

**Table 3:** Eye durations (proportions) of the Leading Eye or the Following Eye in viewing categories FV or NV.

Line	Eye role	Viewing category	Right Approaching Threat	Left Approaching Threat	ANOVA results
1	**Leading Eye**	**FV**	0.73±0.024	0.873±0.023	F_(1,13)_ = 22.585, p<0.002
2	**Leading Eye**	**NV**	0.075±0.016	0.023±0.007	F_(1,13)_ = 9.098, p = 0.02
3	**Following Eye**	**FV**	0.514±0.081	0.32±0.054	F_(1,13)_ = 7.944, p = 0.03
4	**Following Eye**	**NV**	0.343±0.086	0.547±0.069	F_(1,13)_ = 6.135, p = 0.056

The durations as a function of threat approach direction. Provided are ANOVA test results.

In the left-biased sub-group on a narrow pole (N = 10), the leading eye spent significantly longer durations in FV compared with the following eye (F_(1,9)_ = 67.155, p<0.003; Table 1, line 10).

#### Duration in PV

No significant difference was found regarding the PV for both the right-biased or the-left biased sub-groups (Table 1, lines 8,11).

#### Duration in NV

In the right-biased sub-group on a narrow pole (N = 14), the leading eyes spent significantly shorter durations in NV than the following eyes (F_(1,13)_ = 32, p<0.003; Table 1, line 9). The interaction of threat approach direction and eye role was significant (F_(1,13)_ = 8.162, p = 0.039). A separate MANOVA, conducted for the leading eyes and the following eyes, showed that the leading eyes spent significantly shorter durations in NV under left-approaching threats than under right-approaching threats (F_(1,13)_ = 9.098, p = 0.02; Table 3, line 2). In contrast, the following eyes spent significantly longer durations in NV under left-approaching compared with right-approaching threats (F_(1,13)_ = 6.135, p = 0.056; Table 3, line 4).

In the left-biased sub-group on a narrow pole (N = 10), the leading eye spent significantly shorter durations in NV than the following eye (F_(1,9)_  = 38.901, p<0.003; Table 1, line 12).

#### Frequency of eye shifts

In the right-biased sub-group on a narrow pole (N = 14), the frequencies of eye shifts were significantly higher under right-approaching threats compared with left-approaching threats (F_(1,13)_ = 6.706, p = 0.044; Table 2, line 3).

No significant differences were found regarding the frequency of eye shifts in the-left bias group (Table 2, lines 2,4).

The results thus show that eye use in the left-biased sub-group was not lateralized, while eye use in the right-biased sub-group was lateralized, yet only under binocular viewing of the threat.

## Discussion

Lateralization is attracting increasing attention and has been approached at different levels from the merely descriptive [8]–[14] through underlying mechanisms [2], [20], [24], ultimate functions [2], [5], ontogeny [26] and evolutionary roots [5], [36], [37]. Distinctly lacking in most studies, however, is an examination of the lateralization of more than one behavioral aspect while performing a specific task. For example if, in a visually guided task, the motor patterns of limb use are lateralized, will the co-occurring patterns of eye use be lateralized as well?

One of the few cases in which lateralization was examined in the simultaneous use of two sensory organs in a specific task is demonstrated in the Blue gourami (*Trichogaster trichopetrus*) [13]. Gouramies use their long, thin pectoral fins as tactile organs. While exploring a novel object, gouramies exhibit lateralization both of fin use and of eye use: There was a clear preference for exploring stimuli with the left fin and the left eye. Such a lateralization might be attributed to lateralization of brain areas associated with tactile information. Alternatively, it could arise from a primary preference of the left eye for viewing novel objects that, in turn, determines a preference of use of the left fin to explore them. That the use of a given fin was closely associated with the ipsilateral eye may have been due to morphological constraints, restricting the fin crossing the body midline. This example differs from the chameleons here, especially because in the former, information was visual and chemical while in the latter, it was only visual.

In humans, the proportion of the population with a same-side preference of hand and foot use is ca. 0.76, while that of opposite-side preference is ca. 0.24. Moreover, the proportion of humans showing same-side preference of eye, hand and foot use is ca. 0.4 [38]. Thus, most humans (ca. 0.6) exhibit opposite eye and limb use preferences. This raises the question of potential advantages of lateralization. Do such advantages occur only if laterality in the use of two (or more) organs is in the same direction? Do ectotherms and birds, having laterally placed eyes, suffer greater disadvantages when the two organs used are of opposite direction of lateralization?

Initially, it was expected that chameleons would not show distinct lateralized responses. This was because of their highly independent eye movements, full optic nerve decussation and relatively minor inter-hemispheric commissures. In contrast to the above, our results [33], [34] demonstrated that eye use and body motion are lateralized at the level of the entire tested group and, furthermore, that there was lateralization of body motion at a sub-group level.

**Figure 4:**
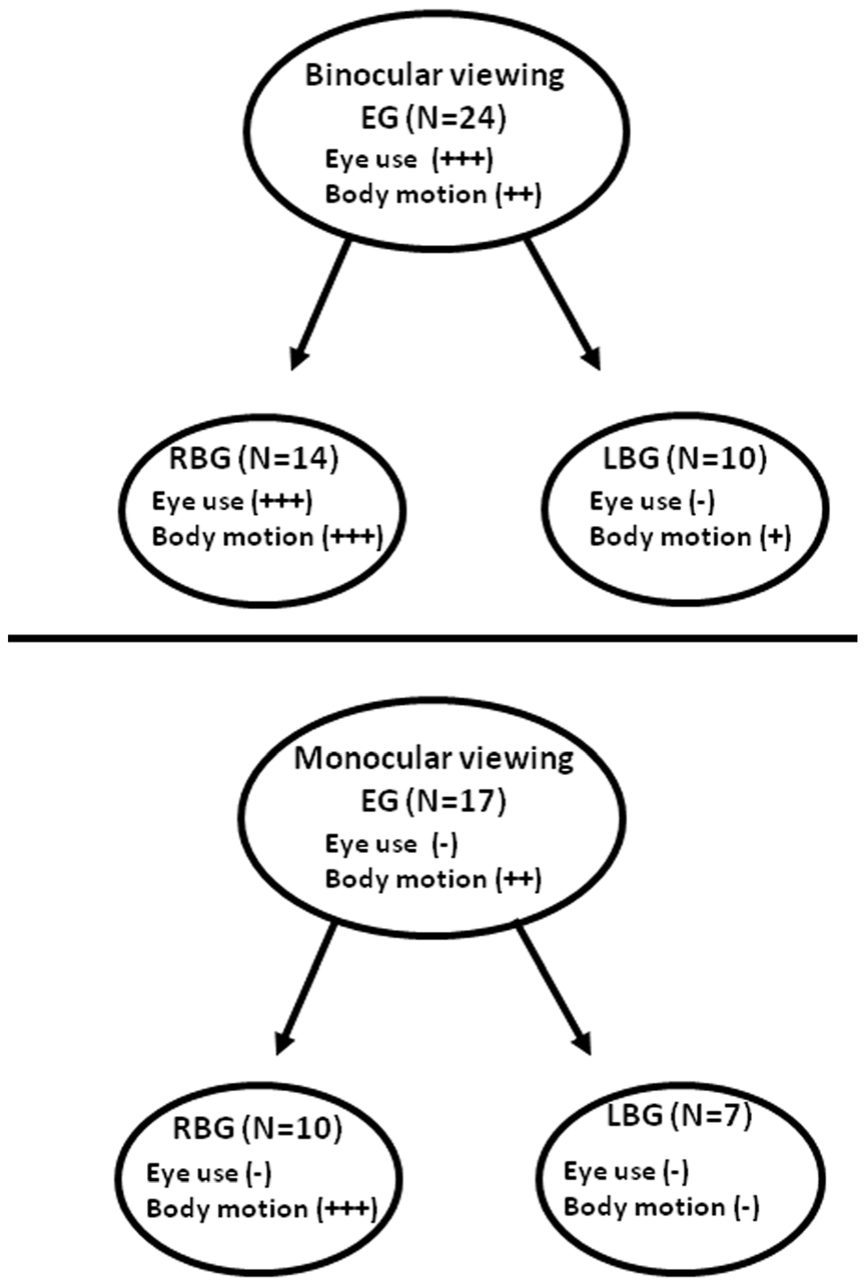
Lateralization of eye use (FV) and of body motion (final exposure) in the tested group and in the sub-groups, under binocular (top) or monocular (bottom) viewing conditions. The plus (+) signs relate to the significance of lateralization (+, p<0.05; ++, p<0.02; +++, p<0.01). Entire group (EG), right-biased sub-group (RBG), left-biased sub-group (LBG).

We here related patterns of eye use to patterns of body motion during an avoidance response (Fig. 4). We found that the right-biased sub-group had a strong lateralization of body motion, expressed in the reaching of final level of exposure (i.e. its ultimate function), and a strong lateralization of eye use in frontal viewing of the threat, under both monocular and binocular conditions. Furthermore, the right-biased sub-group was the determinant factor in the observed lateralization of the entire group. In contrast, the left-biased sub-group showed a relatively weak lateralization of body motion, and only under binocular viewing and no lateralization of eye use under monocular or binocular conditions.

**Figure 5:**
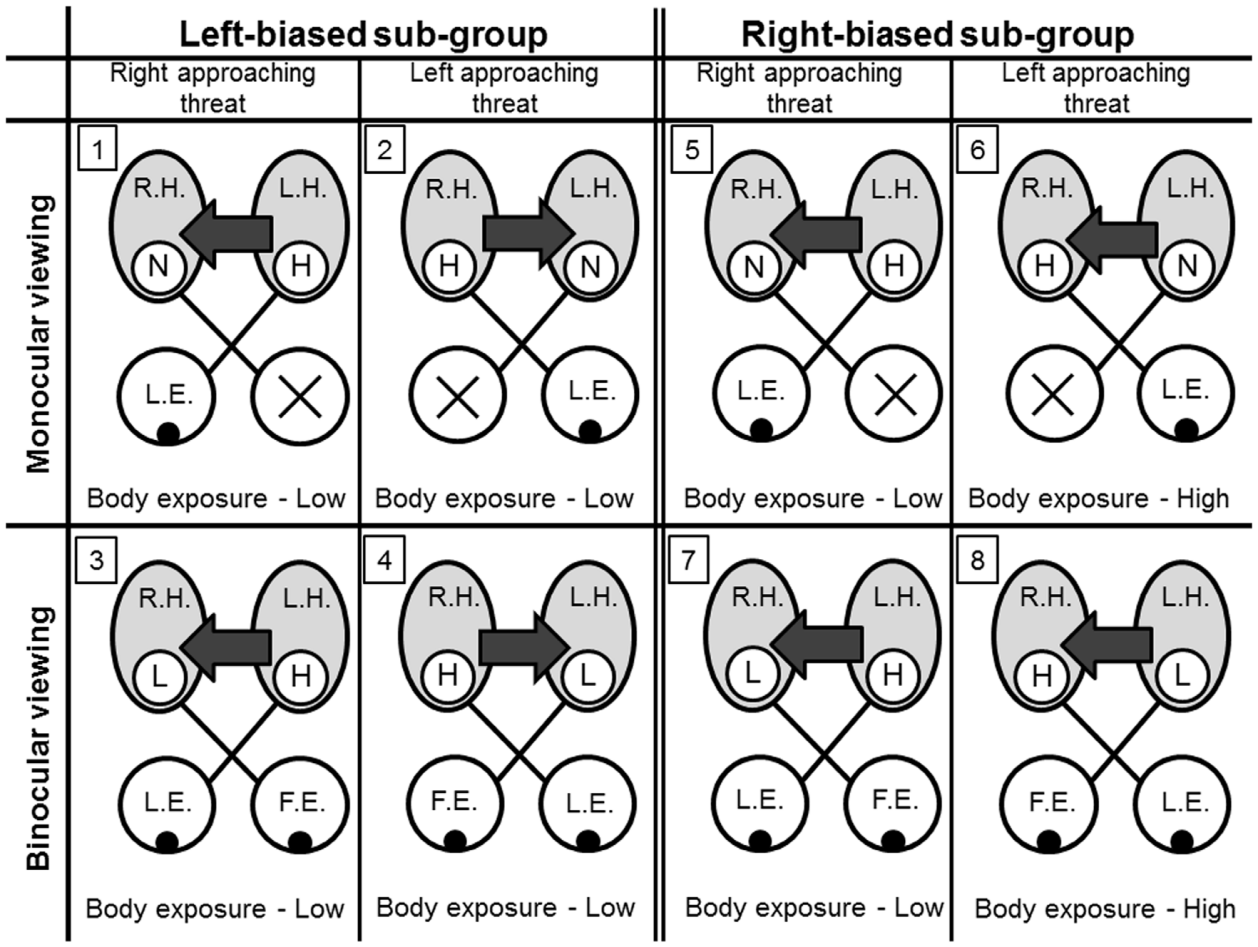
A model of hemispheric dominance relationships and the resulting levels of body exposure, during avoidance behavior, in the two side-biased sub-groups. For a given hemisphere (R.H. – right hemisphere, L.H. – left hemisphere, light gray), the relative excitation is high (H) low (L) or none (N), as a function of eye role (L.E. –leading, F.E. – following). The direction of the arrows depicts the direction of inter-hemispheric dominance. Eyes marked with an X could not view the threat due to the visual obstruction by the relatively wide pole.

These results may indicate that binocularity, in the right-biased sub-group, is not advantageous as it does not ensure the best concealment. In contrast, in the left-biased sub-group, there was no indication of a detrimental effect of binocularity as both sub-groups achieve similar low levels of concealment. Furthermore, the results may point to an interplay in dominance between the brain hemispheres (Fig. 5). We may assume that, for the left-biased sub-group (Fig. 5.1–5.4), the two hemispheres are similar in their capacity to perform motor corrections and the eyes are similar in their use. However, the “leading hemisphere” (the hemisphere connected to the leading eye and thus of higher excitation) is dominant over the contralateral (“following”) hemisphere. In other words, inter-hemispheric dominance is interchangeable. For the right-biased sub-group (Fig. 5.5–5.8), we may assume that the left hemisphere is dominant over the right hemisphere, irrespective of its role as “leading” and thus its level of excitation. Consequently, better body positional corrections are performed under right-approaching threats (Fig. 5.5 & 5.7) while under left-approaching threats (Fig. 5.6 & 5.8), the effect of the left hemisphere is detrimental.

In mammals, limbs are controlled by their respective contra-lateral hemispheres while visual output from each eye is provided to both hemispheres. This may underlie the fact that handedness and eye dominance in mammals, are not regarded as correlated [38], [39]. This is further supported by their patterns of lateralization: in humans, lateralization of hand use has an asymmetric distribution, with roughly three-quarters of any given population being right-handed. The distribution of eye dominance is more symmetrical, with close to one half of the population having a right-eye preference [38]. Experiments relating human handedness to eye dominance indicate that right-handed individuals, with right-eye dominance, are faster to respond in tasks that require reaching for an object. To the best of our knowledge, no such experiments have been reported for other vertebrates.

Several models have been put forward to explain the relationships between eye dominance and handedness in humans, among them: (1) the “No association model” states that there is no association between the two lateralizations, (2) the “Phenotype association model” states that eye dominance is caused by handedness, or that eye dominance is secondary to cerebral lateralization of language, (3) the “Genotype association model” states that genes linked to handedness and language affect eye dominance, and (4) the “Genotypic-phenotypic association model” states that the dependence of eye-dominance phenotype on handedness phenotype is, itself, contingent on the genotype of the individual [40]. Bourassa et al. [40] concluded that there is no single, adequate model for the relationship of eye dominance and hand dominance in humans.

The results here show that lateralization of two aspects, co-occurring in a given response, is complex. Interestingly, the sub-group that showed no lateralization in eye use and in body motion demonstrated a better capacity of concealment from threat, irrelevant of its approach direction. This suggests that un-lateralized individuals may be better adapted to their habitat in which a predator may appear with equal probability from any direction. It remains open whether the observed complexity stems from the chameleons' unique visual system and if, indeed, chameleons may be considered as a model of other ectotherms. While some features, such as full optic nerves decussation, are common to all ectotherms, the large amplitude, independent eye movements is a most uncommon trait. At this stage the comparison with endotherms may be premature and further studies are required on lateralization of several co-occurring behavior patterns.

## References

[1] Bisazza A, Facchin L, Pignatti R, Vallortigara G (1998) Lateralization of detour behaviour in poeciliid fish: The effect of species, gender and sexual motivation. Behavioural Brain Research 91: 157–164. 9578448 10.1016/s0166-4328(97)00114-9

[2] Vallortigara G (2000) Comparative neuropsychology of the dual brain: A stroll through animals' left and right perceptual worlds. Brain and Language 73: 189–219. 10856174 10.1006/brln.2000.2303

[3] Bisazza A, Cantalupo C, Robins A, Rogers LJ, Vallortigara G (1997) Pawedness and motor asymmetries in toads. Laterality: Asymmetries of Body, Brain and Cognition 2: 49–64. 10.1080/713754252 15513053

[4] Randler C, Braun M, Lintker S (2011) Foot preferences in wild-living ring-necked parakeets (*Psittacula krameri*, psittacidae). Laterality 16: 201–206. 20521200 10.1080/13576500903513188

[5] Vallortigara G, Chiandetti C, Sovrano VA (2011) Brain asymmetry (animal). Wiley Interdisciplinary Reviews: Cognitive Science 2: 146–157. 26302006 10.1002/wcs.100

[6] Reddon AR, Balshine S (2010) Lateralization in response to social stimuli in a cooperatively breeding cichlid fish. Behavioural Processes 85: 68–71. 20547214 10.1016/j.beproc.2010.06.008

[7] Malashichev YB, Wassersug RJ (2004) Left and right in the amphibian world: Which way to develop and where to turn? BioEssays 26: 512–522. 15112231 10.1002/bies.20036

[8] Hews DK, Castellano M, Hara E (2004) Aggression in females is also lateralized: Left-eye bias during aggressive courtship rejection in lizards. Animal Behaviour 68: 1201–1207.

[9] Zucca P, Sovrano VA (2008) Animal lateralization and social recognition: Quails use their left visual hemifield when approaching a companion and their right visual hemifield when approaching a stranger. Cortex 44: 13–20. 18387527 10.1016/j.cortex.2006.01.002

[10] Nedellec-Bienvenue D, Blois-Heulin C (2005) Eye preferences in red-capped mangabeys. Folia Primatologica 76: 234–237. 10.1159/000086026 16088192

[11] Dadda M, Koolhaas WH, Domenici P (2010) Behavioural asymmetry affects escape performance in a teleost fish. Biology Letters 6: 414. 20089537 10.1098/rsbl.2009.0904 PMC2880054

[12] Csermely D, Bonati B, Romani R (2010) Lateralisation in a detour test in the common wall lizard (*Podarcis muralis*). Laterality: Asymmetries of Body, Brain and Cognition 15: 535–547. 10.1080/13576500903051619 19739021

[13] Bisazza A, Lippolis G, Vallortigara G (2001) Lateralization of ventral fins use during object exploration in the blue gourami (*Trichogaster trichopterus*). Physiology & Behavior 72: 575–578. 11282142 10.1016/s0031-9384(01)00417-6

[14] Bisazza A, Pignatti R, Vallortigara G (1997) Detour tests reveal task-and stimulus-specific behavioural lateralization in mosquitofish (*Gambusia holbrooki*). Behavioural Brain Research 89: 237–242. 9475631 10.1016/s0166-4328(97)00061-2

[15] Sovrano V, Rainoldi C, Bisazza A, Vallortigara G (1999) Roots of brain specializations: Preferential left-eye use during mirror-image inspection in six species of teleost fish. Behavioural Brain Research 106: 175–180. 10595433 10.1016/s0166-4328(99)00105-9

[16] De Santi A, Sovrano V, Bisazza A, Vallortigara G (2001) Mosquitofish display differential left-and right-eye use during mirror image scrutiny and predator inspection responses. Animal Behaviour 61: 305–310.

[17] Sovrano VA, Bisazza A, Vallortigara G (2001) Lateralization of response to social stimuli in fishes: A comparison between different methods and species. Physiology & Behavior 74: 237–244. 11564473 10.1016/s0031-9384(01)00552-2

[18] Robins A, Lippolis G, Bisazza A, Vallortigara G, Rogers LJ (1998) Lateralized agonistic responses and hindlimb use in toads. Animal Behaviour 56: 875–881. 9790698 10.1006/anbe.1998.0877

[19] Malashichev YB (2006) One-sided limb preference is linked to alternating-limb locomotion in anuran amphibians. Journal of Comparative Psychology 120: 401. 17115861 10.1037/0735-7036.120.4.401

[20] Deckel AW, Jevitts E (1997) Left vs. right-hemisphere regulation of aggressive behaviors in *Anolis carolinensis*: Effects of eye-patching and fluoxetine administration. Journal of Experimental Zoology Part A: Comparative Experimental Biology 278: 9–21.

[21] Bonati B, Csermely D, Romani R (2008) Lateralization in the predatory behaviour of the common wall lizard (*Podarcis muralis*). Behavioural Processes 79: 171–174. 18703120 10.1016/j.beproc.2008.07.007

[22] Pratt DW (1982) Saccadic eye movements are coordinated with head movements in walking chickens. Journal of Experimental Biology 97: 217–223. 7086341 10.1242/jeb.97.1.217

[23] Stamp Dawkins M (2002) What are birds looking at? head movements and eye use in chickens. Animal Behaviour 63: 991–998.

[24] Prior H, Wiltschko R, Stapput K, Güntürkün O, Wiltschko W (2004) Visual lateralization and homing in pigeons. Behavioural Brain Research 154: 301–310. 15313017 10.1016/j.bbr.2004.02.018

[25] Izawa EI, Kusayama T, Watanabe S (2005) Foot-use laterality in the japanese jungle crow (*Corvus macrorhynchos*). Behavioural Processes 69: 357–362. 15896533 10.1016/j.beproc.2005.02.001

[26] Baciadonna L, Zucca P, Tommasi L (2010) Posture in ovo as a precursor of footedness in ostriches (*Struthio camelus*). Behavioural Processes 83: 130–133. 19815058 10.1016/j.beproc.2009.09.004

[27] McGavin S (2009) Footedness in north island kākā (*Nestor meridionalis septentrionalis*). Notornis 56: 139–143.

[28] Randler C, Braun M, Lintker S (2011) Foot preferences in wild-living ring-necked parakeets (psittacula krameri, psittacidae). Laterality 16: 201–206. 20521200 10.1080/13576500903513188

[29] Ott M (2001) Chameleons have independent eye movements but synchronise both eyes during saccadic prey tracking. Experimental Brain Research 139: 173–179. 11497059 10.1007/s002210100774

[30] Harkness L (1977) Chameleons use accommodation cues to judge distance Nature. 267: 346–349. 10.1038/267346a0 865631

[31] Ott M (2006) Visual accommodation in vertebrates: Mechanisms, physiological response and stimuli. Journal of Comparative Physiology A: Neuroethology, Sensory, Neural, and Behavioral Physiology 192: 97–111. 10.1007/s00359-005-0049-6 16172892

[32] Bennis M, Repérant J, Rio J, Ward R (1994) An experimental re-evaluation of the primary visual system of the European chameleon,*Chamaeleo chameleon* . Brain, Behavior and Evolution 43: 173–188. 10.1159/000113633 8193909

[33] Lustig A, Ketter-Katz H, Katzir G (2012) Threat perception in the chameleon (*Chamaeleo chameleon*): Evidence for lateralized eye use. Animal Cognition 15: 609–621. 22460630 10.1007/s10071-012-0489-7

[34] Lustig A, Ketter-Katz H, Katzir G (2012) Visually guided avoidance in the chameleon (*Chamaeleo chameleon*): Response patterns and lateralization. PloS One 7: e37875. 22685546 10.1371/journal.pone.0037875 PMC3369868

[35] Avni O, Baum T, Katzir G, Rivlin E (2010) Recovery of 3D animal motions using cameras and mirrors. Machine Vision and Applications: 1–10.

[36] Vallortigara G, Rogers LJ (2005) Survival with an asymmetrical brain: Advantages and disadvantages of cerebral lateralization. Behavioral and Brain Sciences 28: 575–589. 16209828 10.1017/S0140525X05000105

[37] Vallortigara G (2006) The evolutionary psychology of left and right: Costs and benefits of lateralization. Developmental Psychobiology 48: 418–427. 16886183 10.1002/dev.20166

[38] Nachshon I, Denno D, Aurand S (1983) Lateral preferences of hand, eye and foot: Relation to cerebral dominance. International Journal of Neuroscience 18: 1–9. 6840974 10.3109/00207458308985872

[39] Porac C, Coren S (1975) Is eye dominance a part of generalized laterality? Perceptual and Motor Skills 40: 763–769. 1178363 10.2466/pms.1975.40.3.763

[40] Bourassa DC, McManus IC, Bryden MP (1996) Handedness and eye-dominance: A meta-analysis of their relationship. Laterality: Asymmetries of Body, Brain and Cognition 1: 5–34. 10.1080/713754206 15513026

